# Humanizing medical care for individuals with autism spectrum disorder and their families: the experience of healthcare support in the Comprehensive Medical Care Unit for individuals with ASD (AMITEA)

**DOI:** 10.3389/fpsyg.2026.1716298

**Published:** 2026-02-11

**Authors:** Antonia San José Cáceres, Mónica Burdeus Olavarrieta, Cristina Vicente, Nieves Monleón, Lourdes Sipos, Laura Serrano, Laura Martín, Esther Vela, Mara Parellada

**Affiliations:** 1 Instituto de Investigacion Sanitaria Gregorio Maranon, Madrid, Spain; 2 Centro de Investigacion Biomedica en Red de Salud Mental, Madrid, Spain; 3 Instituto de Salud Carlos III, Madrid, Spain; 4 Universidad Internacional de La Rioja, Logroño, Spain; 5 Instituto de Psiquiatría y Salud Mental, Hospital General Universitario Gregorio Marañón, Madrid, Spain; 6 Universidad Francisco de Vitoria, Pozuelo de Alarcón, Spain; 7 Facultad de Medicina, Universidad Complutense de Madrid, Madrid, Spain

**Keywords:** autism spectrum disorder (ASD), healthcare accessibility, humanization of care, patient narratives, specialized healthcare services

## Abstract

**Introduction:**

Autism spectrum disorder (ASD) is a neurodevelopmental condition frequently associated with comorbidities and high support needs, posing significant challenges for the provision of patient-centered and humanized healthcare. Specialized healthcare programs have been developed to address these needs, yet evidence on user experiences within such models remains limited.

**Methods:**

This qualitative study explored the experiences of patients with ASD, caregivers, and healthcare professionals involved in AMITEA, a specialized public healthcare program in Madrid, Spain. Three focus groups (*n = 24*) were conducted following the Picker model of patient-centered care. Data were analyzed using a hermeneutic phenomenological approach, in accordance with COREQ guidelines.

**Results:**

Participants reported several strengths of the AMITEA program, including respectful and personalized care, effective communication, emotional support, coordination across hospital services, and environmental adaptations tailored to sensory needs. Identified limitations included insufficient coordination beyond the specialized unit, limited resources, challenges during the transition to adulthood, barriers in referral processes, and a lack of ASD-specific training among professionals outside the program.

**Discussion:**

The findings highlight the value of specialized, patient-centered approaches for individuals with ASD, emphasizing the importance of personalized support, adapted environments, and professional training. Extending these practices beyond specialized units may contribute to improved equity, continuity, and humanization of care across healthcare systems.

## 1 Introduction

Autism Spectrum Disorder (ASD) is a neurodevelopmental condition defined as a combination of persistent impairments, across different contexts, in communication and social interaction, as well as restrictive, repetitive and stereotyped patterns of behavior, including sensory difficulties and difficulties in integrating different senses. Causing significant impairment in social, occupational, or other important areas of current functioning is a key criterion for diagnosis (American Psychiatric Association, 2013). These manifestations present themselves in a heterogeneous manner depending on the person’s level of cognitive and adaptive functioning, forming a broad spectrum ranging from mild to severe forms of the disorder (De Groot and Van Strien, 2017). Their need for care is variable and, in some cases, extensive and persistent throughout life, and early identification can help to initiate interventions aimed at improving skills or monitoring relevant clinical aspects. Therefore, it is currently considered essential to promote early diagnostic processes based on multidimensional and comprehensive assessments, covering the entire developmental history along with current assessment (Falkmer et al., 2013; Hayes et al., 2018). In etiological terms, ASD has a predominantly genetic basis, with high variability in the associated influencing factors (Grove et al., 2019; Havdahl et al., 2021; Huguet et al., 2016; Satterstrom et al., 2020), and it is associated with a higher somatic and psychiatric comorbidities rate than the population without ASD [e.g., epilepsy, intellectual disability and learning disorders, anxiety, sleep disorders, etc. (Khachadourian et al., 2023)].

In relation to global rates, the worldwide prevalence of the ASD has shown a progressive and significant increase in recent years (Russell et al., 2022). Currently, the World Health Organization estimates that 1 in 127 of the general population meets the diagnostic criteria for ASD (World Health Organization, 2025). However, overall prevalence rates indicate considerable variability between regions and studies, with ranges fluctuating between 0.01 and 4.36% (Zeidan et al., 2022), which is the result of substantial differences in diagnostic methods, inclusion and exclusion criteria, and/or contextual factors, such as access to services and the level of public awareness, among others (Talantseva et al., 2023). In this context, ASD poses a significant challenge to health care and social protection systems worldwide. The clinical complexity of ASD, together with the continuous need for therapeutic and community support for individuals on the spectrum and their families, underscores the necessity of comprehensive public policies and specialized resources that guarantee early intervention and sustained follow-up across the life course, thereby ensuring effective, person-centered care (Salari et al., 2022).

In this context, contemporary health intervention models increasingly recognize the importance of approaches grounded in personalization and patient-centered support (Busch et al., 2019; Cano et al., 2023; Tripodi et al., 2017). Such perspectives promote more empathetic and holistic care experiences (Borges et al., 2025; de la Fuente-Martos et al., 2018). A clear example is provided by person-centered care models, which advocate for a paradigm shift away from the traditional biomedical framework. These models emphasize a more collaborative therapeutic relationship, where the patient and their life context are placed at the forefront of intervention planning and implementation (Rathert et al., 2013). Within this setting, family-centered approaches have gained recognition in efforts to improve healthcare for autistic individuals. These approaches are grounded in collaboration between families and professionals, shared decision-making, and consideration of families’ knowledge, and are integrated in practices such as individualized care planning, adapted clinical pathways, and specialty services. Previous studies suggest that family-centered interventions are associated with better communication, greater caregiver confidence and satisfaction, and improved continuity of care, as well as reduced stress and barriers when navigating healthcare systems (Nicholas et al., 2020; Zhang and Wang, 2025).

A multidisciplinary clinical approach is essential in the case of neurodevelopmental disorders, as it allows professionals from different fields, such as psychiatry, psychology, neurology, nursing, speech therapy, and/or occupational therapy, to coordinate their work in order to provide comprehensive and effective responses (LaFrance et al., 2019; Strunk et al., 2017). However, numerous barriers persist in contemporary health systems that hinder equitable and effective access to general health services for people with ASD (Doherty et al., 2020), mainly in three critical areas: equal access to medical care, the availability of adequate services during transitions between life stages, and advances in research and development of specialized health programs (Malik-Soni et al., 2022), contributing to deteriorating health and increased family stress (Almasoud and Alqahtani, 2023). Particularly concerning is the lack of coordination across different levels of care, as well as the heterogeneity in service provision, both of which undermine continuity of care. This issue is especially critical in disorders that demand constant and adaptable interventions throughout the various stages of the life cycle (Gabovitch and Curtin, 2009; Shamash and Hinman, 2022). Compensating this, the emergence of personalized healthcare programs for people with ASD that implement individualized healthcare intervention plans in coordination with different professional and social agents is becoming established as an essential best practice for optimal social and healthcare support (Ip et al., 2019), not only to improve healthcare systems, but also to promote a cultural shift in global policies and strategies that recognize neurodiversity as an inherent expression of the human condition (Heraty et al., 2023; Wallace et al., 2012).

In response to this paradigm shift, there is a growing trend toward recognition of ASD in the healthcare setting, along with an appreciation of the positive effects this entails, particularly in terms of higher-quality service delivery and a better patient experience (Doherty et al., 2021). For all these reasons, it is increasingly common to find specific healthcare initiatives tailored to the particular needs of people with ASD, in which specialized programs are designed and implemented to respond more effectively and individually to their needs (C G Davenport et al., 2025; Kim et al., 2019; Nygren et al., 2025; Thompson and Tielsch-Goddard, 2014).

In response to this need, a specific healthcare program for people with ASD was launched in 2009 in Madrid (Spain): the Integral Healthcare Unit for People with Autism Spectrum Disorder (AMITEA) at the Gregorio Marañón University General Hospital (Parellada et al., 2013). The program was established under a collaboration agreement between the Regional Ministry of Health of the Community of Madrid and Federación Autismo, a coalition of associations representing families of people with autism. Over the previous 10 years, these associations had advocated with the Ministry for the creation of a public service to address the healthcare needs of their relatives with autism. This unit provides specialized medical care for ASD to people in the region of Madrid. It is made up of a team of professionals who are experts in ASD, including psychiatrists, psychologists, two specialist nurses, a neurologist, a gastroenterologist, and a social worker, with varying levels of dedication to this unit. Since its inception, the population it serves has grown annually to more than 4,000 patients in their books. AMITEA is structured around multiple key elements that are essential to its operation: facilitation and coordination of the clinical process (in terms of access, appointments and referrals, grouping of hospital visits, etc.), physical accompaniment in specialist consultations, prioritization and specific adaptations (e.g., environmental and temporal anticipation, facilitated communication), individualized management of each case and continuity of care, professionalization of care (e.g., adapted nursing procedures, clinical consensus sessions, etc.), and specific training. In this way, the scope of AMITEA transcends the care provided directly to these patients at its facilities and extends indirectly to other hospital services. The target population includes all individuals, regardless of age, living in the Region of Madrid with a diagnosis of ASD and a need for specialized medical care. Since the inception of AMITEA, efforts have been made to train both primary care and specialized physicians in the region in managing the physical and mental health needs of the ASD population. These efforts, combined with the increasing demand on AMITEA, have guided the development of the service into a more specialized unit, continuously addressing medical issues that require hospital-level care. Less severe medical or psychiatric problems are increasingly managed within the community, in line with the care provided to other patients within the national health system, which is tax-funded and universal. This specific care unit is unique at the national level, and for 16 years, it was responsible for the specialized care of ASD at the hospital level across the entire Madrid region (population of 6,000,000). Following this successful enterprise, a parallel unit was opened in Getafe (Southern Madrid) in 2022, in coordination with AMITEA-Marañón, with the aim of sectorizing care and strengthening proximity-based services. Furthermore, AMITEA-Marañón is part of the Institute of Psychiatry and Mental Health of the Hospital and shares its commitment to pursuing excellence and the continuous improvement of all clinical, research, and management processes.

Since the inception of this unit, after accumulated clinical experience and in light of social changes occurred in the last years, including the COVID-19 pandemic, we consider that the needs and expectations of our patients and their families may have evolved. Therefore, a review of the service is needed to ensure ongoing adaptation and keep on providing adequate and person-centered care. Within this framework, the present study offers a novel, user-informed qualitative evaluation of a long-standing autism specialty care unit, conducted sixteen years after its implementation. We aim to delve deeper into the experiences of ASD patients themselves, their primary caregivers, and the social and healthcare professionals linked to this service, and thus to study and understand the changing needs of this population to ultimately adapt to better patient-centered practices and serve as a model to other public institutions. Specifically, this study seeks to explore how care is currently experienced across key dimensions of humanized and person-centered healthcare and identify perceived strengths and areas for improvement in the organization and delivery of care, within a public healthcare setting.

## 2 Materials and methods

### 2.1 Study design

This study adopts a descriptive qualitative approach aimed at gaining an in-depth understanding of the perceptions and experiences of the different actors involved in the clinical care service provided by the AMITEA unit. In order to ensure methodological rigor, transparency of the research process, and the credibility and potential replicability of the findings, the standardized COREQ (Consolidated Criteria for Reporting Qualitative Research) (see Supplementary Data Sheet 1) guidelines proposed by Tong et al. (2007) are used as a reference. This guide, consisting of 32 items, allows for a critical evaluation of three fundamental dimensions in qualitative studies: (1) the characteristics of the research team and its relationship with participants; (2) the study design; and (3) the analysis and presentation of results. This methodological design seeks to capture the complexity inherent in the subjective experiences of people with ASD, their primary caregivers, and social and healthcare professionals in relation to the care received/provided, the care processes, and the perceived impact of the specialized intervention of a specific care unit for people with ASD.

### 2.2 Participants and sampling

The study sample consisted of a total of 24 participants, divided into three groups according to their relationship with the AMITEA program: (1) six adults diagnosed with ASD, active users of the AMITEA unit; (2) eight primary caregivers (family members) of two children and six adults diagnosed with ASD who were treated at the service; and (3) ten professionals from the social and healthcare field with different clinical and care profiles, linked to the service either directly (AMITEA program personnel) or indirectly (personnel from units that receive consultations or referrals from AMITEA professionals). Table 1 shows the main characteristics of the participants for each group.

**TABLE 1:** Sociodemographic characteristics of the participants in the three focus groups.

Sociodemographic characteristics	Patients (*n* = 6)	Caregivers (*n* = 8)*		Professionals (*n* = 10)**
		Caregivers	Their cared one with ASD	
Sex (Male: Female)	5:1	2:6	6:2	1:9
Age (in years)	22.7 (*3.7*) [19, 29]	57.6 (*13.2*) [45, 84]	26.1 (*13.5*) [10, 52]	46.2 (*10.0*) [36, 58]
Time included in AMITEA (in years)	8.2 (*5.2*) [3, 15]	7.9 (*5.4*) [2, 16]		6.1 (*4.66*) [2, 16]
**Education**				
High school	2	1		0
Vocational training	2	0		0
Graduate degree	2	2		1
Postgraduate/PhD	0	5		8
**Employment status**				
Student	4	0		0
Employed	1	4^†^		9
Unemployed/seeking	1	1		0
Retired	0	2		0
Full-time carer	0	1		0
**Referral to AMITEA from**				
(Child) Physician	5	5		–
Psychiatrist	1	2		–
Unsure	0	1		–
**Specific training on ASD**				
Formal	–	–		1
Non-formal	–	–		6
None	–	–		3
**Link to AMITEA’s services**				
Internal	–	–		4
External frequent	–	–		2
External sporadic	–	–		4

*Two parents from the same child.

** Professional specialties: Psychiatrist (*n* = 3), social worker, advanced-practice nurse, psychologist, neurologist, pediatrics ICU resident, dermatologist, pediatrics nurse.

† 2 full time, 2 part time.

**FIGURE 1:**
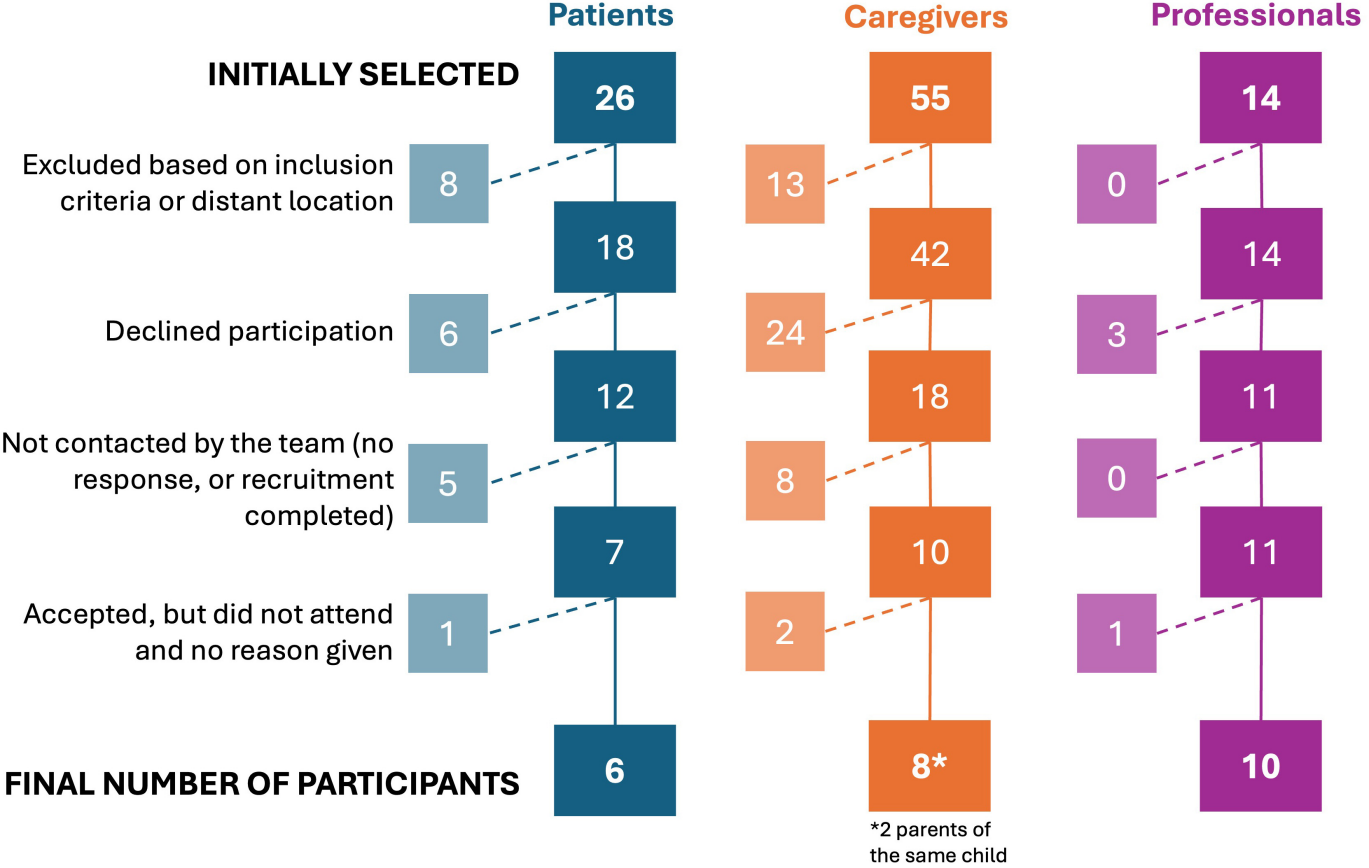
Flowchart describing the recruitment and selection process for the three groups of participants. “Initially selected” indicates the pool of individuals that could potentially be included, based on *a priori* screening of eligibility criteria.

Participants in groups 1 and 2 were selected through purposive sampling from all medical records in the service, which allowed researchers to deliberately identify and contact individuals who met the defined eligibility criteria (for group 1: functional verbal communication skills and clinical stability; for group 2: representativity in terms of clinical characteristics, sex and age of the person with ASD). These characteristics were considered essential to ensure an accurate representation of the service users, as well as for relevance, depth, and significance of the information obtained. Potential participants of each group were pre-selected and contacted by phone or email, and recruitment was discontinued once each group reached 8 to 10 confirmed participants. As a result, individuals who declined participation or were not contacted due to recruitment completion did not systematically differ from included participants on these characteristics. To minimize selection bias, the researchers had no clinical contact with the participants and were unaware of their opinions about the service, but ensured that group 1 consisted of users diagnosed with ASD with functional verbal communication skills and clinical stability compatible with the research process (i.e., participants were not hospitalized, and were not experiencing psychiatric decompensation or severe aggression). In group 2, priority was given to maintain representative percentages of the service users in terms of their clinical and functional characteristics of the ASD (i.e., high and low support needs), age ranges (below or equal and above 18 years of age) and sex ratios, as well as the length of time the patient had been linked to AMITEA, with the aim of collecting a representative sample of different experiences and needs. Finally, group 3, composed of professionals from the social and healthcare field, included those with direct or indirect professional links to the service, seeking to represent different disciplinary profiles and levels of care responsibility. Figure 1 shows the selection and recruitment process, including the initial number of people contacted and the final number of participants included in the focus groups. For unknown reasons, one to two individuals in every group that agreed to participate did not attend on the given day.

### 2.3 Data collection

Data collection was carried out by conducting three focus groups (one for each of the groups participating in the study) on separate days. The sessions were held in person at the facilities of the Institute of Psychiatry and Mental Health at Gregorio Marañón Hospital, with an average duration of 93 min per meeting.

Each focus group was moderated by one of the researchers on the team, who played an active and direct role in leading the dialogue. The moderator was a researcher with experience in autism and an interest in family-centered healthcare, who was not involved in the direct clinical care of participants. Participants in groups 1 and 2 had no prior relationship with the moderator; four participants in group 3 were acquainted with the moderator in a professional context. The moderator was supported by a second researcher responsible for taking notes, which were displayed in real time to participants on a whiteboard, without intervening in the group dynamics. In addition, a third researcher acted as an external observer, refraining from participating in the sessions but recording field notes and complementary observations on paper. The sessions were recorded using two voice recording devices, ensuring the accuracy of the information captured for subsequent analysis. These transcripts were not shared with participants.

The interview script used to guide the focus group discussions was designed based on Picker’s Eight Principles of Person-Centered Care (see Supplementary Data Sheets 2, 3), which constitute an ethical and methodological framework for evaluating and improving the quality of care from the patient’s perspective. These principles include: (1) respect for patients’ values, preferences and expressed needs, (2) coordination and integration of care, (3) information and education, (4) physical comfort, (5) emotional support and alleviation of fear and anxiety, (6) involvement of family and friends, (7) continuity and transition, and (8) access to care (Pickler Institute, 2025). The adoption of the Picker principles not only provides ethical and methodological guidance for person-centered care but also allows for a sensitive and rigorous approach to the complexity inherent in therapeutic support for chronic health conditions (Loban et al., 2025) which enabled the design of a consistent script for gathering quality information aligned with the study’s objectives.

### 2.4 Ethical considerations

The study was conducted in accordance with the principles of the Declaration of Helsinki and approved by the Gregorio Marañón Hospital Ethics Committee (approval reference *AMITEA Acompaña*). All participants provided written informed consent prior to participation in the study, in accordance with the ethical principles established in the Declaration of Helsinki (World Medical Association, 2025). The consent process was carried out individually, ensuring that each participant fully understood the objectives, procedures, risks, and potential benefits of the research, as well as the voluntary nature of their participation and the possibility of withdrawing at any time without repercussions.

### 2.5 Data analysis

The data analysis was carried out by the research team, who had previous experience in qualitative studies and care for people with ASD and their primary caregivers. The research was conducted from a qualitative perspective, based on Paul Ricoeur’s hermeneutic phenomenology, as this is considered an effective research strategy based on establishing a dialectic between explanation and understanding that allows for an in-depth interpretation of the meaning that subjects attribute to their experiences (Ricoeur, 1976; Ricoeur, 1981). According to Ricoeur, textual interpretation is understood as a dialectical process composed of two complementary movements. The first moves from understanding—defined as “a naïve grasping of the meaning of the text as a whole”—to explanation; the second moves from explanation to comprehension, understood as “a sophisticated mode of understanding supported by explanatory procedures.” Drawing on this theoretical framework, several scholars have developed hermeneutic phenomenological methodologies for the qualitative analysis of lived experience. Although these approaches differ in their specific procedures, they all adhere to Ricoeur’s dialectic through three successive phases: an initial, surface-level interpretation; a structural analysis; and a final, in-depth interpretation of the text. These phases correspond to the stages outlined by Ricoeur. Within this analytic process, data saturation was considered achieved when successive focus groups and coding iterations no longer generated new meaning units, themes, or interpretive insights relevant to the research questions, and when additional data served only to confirm previously identified structures of meaning. In this work, we have structured the analysis of the text in the same three phases, reflecting the so-called hermeneutic arc and performing the following actions in each of them:

• Phase 1 (Naïve reading):

The authors conducted an initial reading of the interview transcripts, aiming to apprehend their overall meaning and to relate it to existing literature as well as to the authors’ own preconceptions. This process allowed for the development of a preliminary interpretation, which was subsequently subjected to validation in the following phases.

• Phase 2 (Structural analysis):

The researchers employed the qualitative data analysis software Atlas.ti v25 software^®^, to identify the structure of meanings inherent in the text. The use of this software facilitated the suspension of the researchers’ preconceptions (epoché), enabling a descriptive explanation of the text. This explanation was then validated against the different initial interpretations by lifting the suspension previously enacted. The validated interpretations were collectively discussed among the researchers and contrasted through a deliberative judgment in order to identify the “most plausible” interpretation.

• Phase 3 (In-depth analysis):

The research team conducted a further joint reading of the interview transcripts, now taking into account the insights derived from the structural analysis. This phase involved the application of a critical perspective that enabled openness to the world to which the text refers.

Two additional concepts in Ricoeur’s thought are essential for understanding his theory of interpretation: distanciation and appropriation, as well as the dialectical interplay that unfolds between them. Without aiming to be exhaustive, distanciation in Ricoeur refers to the separation between the author of the text and the work itself, whereas appropriation constitutes the culminating moment of hermeneutics, in which the reader opens themselves to the world of the text, integrating it into their own way of being-in-the-world. Understanding a text entails the overcoming of distanciation and, at the same time, presupposes its very existence in order to occur.

With regard to our analysis, the transcription of the interviews into a written medium ensures the establishment of distanciation, which would not occur within the dialogical framework of the face-to-face interview itself. Appropriation, in turn, emerges as the outcome of the methodological process of interpretation carried out by the research team. Notably, within the text generated from the interviews, three distinct “worlds” can be identified, one corresponding to each of the participant groups. Consequently, although both the naïve reading and the structural analysis were conducted in a unified manner, the final critical reading resulted in a separate interpretation for each reference group.

### 2.6 Trustworthiness and rigor

To ensure the reliability and methodological rigor of the study, the criteria for reliability and rigor in qualitative research proposed by Lincoln and Guba (1985) were adopted. The credibility of the study is ensured by the faithful transcription of the interviews, allowing for an accurate representation of the experiences narrated, as well as by the triangulation of the data obtained by both the research team members and the researchers. Transferability is demonstrated through a rich and detailed description of both the context and the participants involved in the study, making it easier for other researchers to evaluate the applicability of the findings to similar settings. Dependency is addressed through a thorough description of the study’s methodological design, as well as the systematic and consistent coding of the data obtained. Finally, confirmability is guaranteed through constant reflection on the part of the research team, coupled with transparency in the analytical and interpretative decisions made during the process. Data triangulation has also made it possible to reduce biases arising from the interpretative involvement of the research team.

## 3 Results

### 3.1 Structural analysis results

Following Ricoeur’s reflexive hermeneutic phenomenology, after the first naïve reading of the interviews a structural analysis of the text was performed using Atlas.ti v25 software^®^, in order to identify the text meanings structure. As a result, data categories emerged from a deductive analysis guided by the eight principles of Picker’s person-centered care model, which allowed the codes to be organized in a manner consistent with key dimensions of healthcare support. Below, we present these core meanings, illustrated with excerpts from the discourse that demonstrate the ethical and symbolic density, but also the emotional intensity of the experiences recounted by patient users (U), family members (F), and professionals (P).

#### 3.1.1 Respect for patients’ values, preferences and expressed needs

Patients with ASD and their families report a highly positive assessment of the AMITEA service, highlighting both the team’s professional expertise and the quality of the clinical relationship established. The individualized follow-up of each case, together with the staff’s respectful, committed, and empathetic approach toward patients and their family contexts, is identified as a key distinguishing feature compared to non-specialized services.

*“I think we have always found ‘something’ in the AMITEA team that is not easy to find in other professional contexts”* (F4).

In response to this individualized care, patients with ASD strongly emphasize the feeling of understanding their condition from professionals, which places them in a subjective position of greater emotional security and acceptance within the clinical environment. This perception of recognition is closely linked to the healthcare team’s ability to adapt its communication methods, clinical practices, and intervention rhythms to the specific characteristics of each user, creating a therapeutic environment of mutual trust and validation of individual experience.

*“The first place where I feel understood and have been understood in psychiatry. Once I started psychiatric follow-up here, I finally became aware that people understood me and understood my language”* (U3).

In this context, the active participation of people with ASD in therapeutic decision-making is key. The testimonies collected highlight the importance of professionals respecting patient autonomy and avoiding coercive practices, especially regarding pharmacological decisions. Dialogic and shared deliberation is presented as a guiding principle of this care model.

*“In my case, it was mainly their respect for changing my medication at a later time, which I preferred”* (U2).

For their part, professionals involved in the service, both directly and indirectly, clearly recognize the added value represented by the existence of a resource specifically designed to address the specific needs of people with ASD and their families. This recognition refers not only to the technical quality of the care service, but also to its ability to facilitate clinical processes.

*“For me, [AMITEA]…I have always understood it as a resource that facilitates medical consultations and support for children and adults with ASD (…). I think the work they do is quite clear, very well-defined, and very good”* (P10).

Professionals from non-specialized services, such as hospital emergency departments or other medical specialty consultations, report high levels of satisfaction and appreciation for the AMITEA service. They emphasize that referrals are typically accompanied by relevant clinical information or by direct support from AMITEA professionals, which facilitates anticipation of patient needs, adaptation of care, and reduction of uncertainty for patients, accompanying persons, and healthcare staff. This prior coordination contributes to a safer and more efficient care environment, enhancing professional preparedness, patient-centered care, and positive perceptions among companions, while reducing clinical risks and critical events, particularly in emergency settings.

In response to having support from AMITEA personnel: *“My feeling from working at the emergency room is one of total gratitude. When you take a medical approach, perhaps with a patient who comes in with behavioral problems, and you actually manage to carry out a physical examination that seems silly, and you can rule out pathologies…, I think we are especially grateful”* (P8).

From the perspective of healthcare professionals, the presence of inherent tensions in clinical encounters is often acknowledged, especially when the expectations of the healthcare team differ from those of families of patients with ASD. While professionals are strongly committed to taking a holistic approach that considers the experiences of both patients and their primary caregivers, gaps frequently emerge between what families expect from services and what those services are realistically able to provide.

*“I think there is a huge discrepancy when it comes to expectations, and a large part of the job is often adjusting expectations of what we can do”* (P2).

#### 3.1.2 Coordination and integration of care

Interprofessional coordination with internal referrals takes on central importance in the provision of truly comprehensive and holistic care. In people with autism, the presence of comorbidities and other associated chronic conditions is highly frequent, which means that patients are continuously exposed to a wide range of clinical experiences. In this context, both interviewed patients and users recognize as key elements the support offered by the unit (especially by AMITEA nursing staff to liaise with other medical specialties), as well as the active involvement of the medical team in establishing coordination links with the various clinical and non-clinical services (e.g., educational or residential) involved in patient care.

*“The psychiatrist asked me, ‘Can we talk to the diversity unit at your university?’ and I said, ‘Of course,’ and I really appreciated that because it meant that the university was more understanding of my needs, and I loved that”* (U3).

Families and primary caregivers emphasize the high level of coordination provided by the service, which they perceive as a distinguishing feature compared with non-specialized services. In these settings, despite operating within social and healthcare systems, difficulties often arise in recognizing the specific needs of patients with ASD, such as preferences for reduced waiting times, accommodations for sensory sensitivities, or adjustments to personal circumstances. These challenges are particularly evident in common care contexts for AMITEA users, including hospital specialist clinics and public mental health centers to which users are referred after discharge, resulting in care pathways characterized by discontinuity and variability in the quality of clinical responses.

*“When they refer you to another hospital (…) what you notice is that they have no idea how to treat them [our children]… And you can see that they are overwhelmed because they don’t know how to handle the situation*” (F8).

All actors involved in the interviews unanimously recognize the work carried out by the specialist nurses. Their knowledge and deep understanding of ASD allows them to play an expanded role in the care process, taking on functions that go beyond conventional care and are articulated in a proactive intervention focused on the individual needs for each patient.

From one of the nurses: *“It’s more than just adapting to AMITEA’s procedures. If the child is on the floor and we have to take blood while they are lying down, the nurse lies down on the floor and takes the blood lying on the floor… If we need to sing a song to the child to calm him/her down or to give him/her a massage, we will do so… we sing, play… I think emotional support is given to meet the needs”* (P4).

However, professionals outside the specialized service also identify persistent barriers that hinder interprofessional coordination and the effectiveness of internal referrals. The high workload, the absence of mechanisms to identify patients as having an ASD diagnosis, the lack of standardized protocols and policies for the care of people with complex needs, and the difficulty in clearly defining the reference professionals within multidisciplinary teams, are named as the factors responsible for compromising the continuity and consistency of the user’s clinical follow-up.

*“I often coordinate directly with pediatricians, for example. But of course, [I need] to find out which pediatrician, in which health center, the reason for the referral, what their needs are…Of course, this effort depends on the willingness and time available of the coordinating professional”* (P2).

Despite the strong individual commitment of professionals in the social and healthcare sectors, challenges in communication, both between specialist and primary care services and between hospital networks and external social and healthcare networks, combined with the absence of clearly designated reference figures within health centers, undermine the continuity of therapeutic processes. These gaps also generate significant uncertainty in the planning and monitoring of care pathways designed specifically for people with ASD.

#### 3.1.3 Information and education

Timely and personalized access to ASD-related information and resources is essential for improving the quality of life of autistic individuals and their family environments. Beyond healthcare needs, autism-related demands often extend into psychoeducational, behavioral, and social domains, requiring additional guidance for patients and caregivers. In this context, data from the three stakeholder groups included in the study reveal differing perceptions regarding the adequacy and accessibility of information within the healthcare process.

From a professional perspective, both formal and informal information-sharing mechanisms are available from the point of diagnosis and throughout the life course of individuals with ASD and their families. Professionals in the social and healthcare sectors also note the increasing informational autonomy of many patients and families, who frequently attend consultations well informed. Accordingly, professionals report access to up-to-date resources that are mobilized either in response to user requests or as specific needs emerge during care.

*“Newly diagnosed patients who still are in the middle of the process, families of very young children, or those grieving the diagnosis… I think that’s where we really provide them with good support (….) and I think we do have a lot of information: about many resources, the steps to follow, referral to specialized services and all these programs…for disability, early intervention…”* (P2).

However, users and primary caregivers report somewhat different perceptions. Families indicate that the information received is not always sufficient to meet their specific needs, particularly regarding daily management and guidance on available external resources. Consequently, they suggest the development of more effective communication and information-dissemination channels, such as periodic newsletters or more functional internal referral systems. While acknowledging the broad availability of resources and information, families note that limitations in open and equitable distribution result in fragmented dissemination and, ultimately, unequal access to services.

*“Maybe they could tell everyone, because it’s impossible for what might interest each person to be within everyone’s reach… Because the psychiatrist is not there with that purpose, she/he could pass on each patient’s interests on to someone in charge…”* (F1).

From the perspective of the users themselves, although they generally acknowledge having received adequate information regarding the reason for their consultation and the clinical aspects addressed therein, they also point out certain shortcomings, notably the limited availability of specific information on diagnosis in adulthood, the absence of referral spaces where they can share experiences with other users in similar situations, and the lack of practical materials that promote autonomous and informed decision-making.

*“I do miss meeting people who have been through the same thing, who suddenly find themselves diagnosed, and it would be nice to know that there are other people who have been through the same thing as me”* (U3).

#### 3.1.4 Physical comfort

Users of the AMITEA program highlight that the physical environment, including the spatial layout and available resources, is well adapted to their needs. The organization of the waiting area into structured “corners,” together with the use of visual and anticipatory supports, contributes to an environment perceived as predictable and organized, which is particularly relevant for patients with ASD. Nonetheless, participants also identify areas for improvement, noting the limited availability of manipulative and multisensory resources that could enhance the waiting experience. Suggested improvements include the installation of water fountains or coolers, the expansion of literary materials in the reading corner, and the review of physical barriers that may hinder communication between administrative staff and patients with ASD, such as the methacrylate screens at reception and admission desks.

*“They could have a Rubik’s cube in the waiting room (…). I go upstairs and ask for a receipt, but I can’t understand the receptionists because there’s a methacrylate screen in between us”* (U6).

Both direct users of the service and their families agree that, beyond physical comfort, the in-person support provided by the specialists nurses represents an added value and differentiating factor of the program. This support allows patients to attend outpatient appointments accompanied by a specialized professional, which increases their sense of security, reduces the uncertainty associated with the clinical environment, and improves their care experience.

*“There is a fundamental aspect that outweighs any inconveniences that may arise, which is the existing support from the very beginning with an accompanying professional. If you go to a crowed place, the child is protected because they are accompanied by specialists,… And they [our children] are being listened to and cared for”* (F4).

Another important point to highlight is that membership of the AMITEA program facilitates the recognition of patients with autism within the hospital’s clinical management system. This recognition allows for automatic prioritization in appointments at various hospital services, reducing waiting times and thus mitigating the stress derived from the sensory and emotional overload associated with these environments.

*“In my experience, and that of other women, they always let us in first (…). My friends, some of whom have children with ASD, come here because their children have a hard time in emergency rooms, and they come here so they can be seen first, because it pops up on the computer”* (F7).

Although users with ASD express a general level of satisfaction with the physical comfort offered, both family members and professionals involved in care point to several areas for improvement. Particularly, there is a need to adapt the physical spaces of services outside the unit (other specialized clinical appointments, emergency rooms, etc.), incorporating resources such as visual aids, regulated sensory stimuli, and acoustically controlled environments that are suitable for patients with specific sensory needs.

*“There’s the issue of adaptation environments with sensory toys, lighting, noise cancellation… There are so many areas for improvement in that regard. And then there’s the emergency room, which is another world altogether… I don’t even know where to start about that one”* (P8).

In line with the above, participating professionals highlight two key areas whose improvement would be essential to consolidate a truly responsive care model for patients with ASD. First, there is a need to establish standardized protocols and policies that enable early identification of these patients when approaching the healthcare system, thus facilitating the adaptation of spaces and clinical interventions from the first contact. Secondly, they emphasize the urgency of promoting specific, cross-disciplinary training in autism for all clinical staff, with the aim of building an informed and competent care culture. These actions, which complement the improvements in the physical and organizational environment mentioned above, are essential for moving toward comprehensive care that is, above all, tailored to the specific needs of patients with ASD and their families.

*“But professionals aren’t aware, and they see children with ASD as weird, like they’re evil… You hear all kinds of shameful things… There are lots of talks on ASD that I try to go to, but what happens? The other professionals, the important ones, don’t go… So, I think there should be mandatory training in certain services so that everyone knows what it means to have a child with ASD… Sometimes I’m amazed at how they [other professionals] treat them and what they say to them… We need to raise awareness amongst professionals who are not aware and who do not want to be aware…there are some who need to be influenced…* (P2).

#### 3.1.5 Emotional support and alleviation of fear and anxiety

Emotional support in the care process is perceived by the different agents involved as a key component in ensuring comprehensive and humanized care for people with ASD and caregivers. From the patients’ perspective, access to certain support channels at critical times is valued positively, as is the close relationship established with healthcare professionals, especially their referring psychiatrists. The possibility of having a direct channel of communication for urgent situations, such as email, is understood to be a very useful resource, as well as a reassuring element, particularly at times of greater emotional vulnerability for them.

*“You shouldn’t abuse it but knowing that you can count on it via email in emergencies is fantastic, in my opinion”* (U3).

However, families have mixed opinions about their emotional support during the care process. They explicitly recognize and value the commitment of the AMITEA unit’s professional team as an important source of relief and psycho-emotional support but define fewer positive experiences especially in services outside the program, which lack of sensitivity. These negative experiences have a significant emotional impact that increases the existing burden and causes unnecessary stress, and families call for a more specialized training in emotional and relational skills, involving the creation of specific resources to adequately address the psychological burden of caring for a person with autism.

*“(I miss) support for the family, because there are situations or bad spells that are really difficult to deal with”* (F6).

From the professional perspective, emotional support is an integral component of daily clinical practice, although its delivery is largely shaped by contextual factors, including family expectations and the nature of the relationship established with users. To address emerging emotional needs, professionals incorporate adaptive strategies into routine care, such as increasing flexibility in the physical environment and adjusting clinical procedures to patients’ sensory and behavioral characteristics, thereby fostering greater safety and trust during interventions. Nevertheless, professionals note that structural constraints within the healthcare system (such as staff shortages, workforce instability, and service saturation) limit the provision of specialized and sustained emotional support over time.

#### 3.1.6 Involvement and family and friends

The active participation of family members and close friends in the care process is generally perceived as a positive and necessary element by the three groups involved in the study.

Patients with autism value the involvement of their primary caregivers, especially when it occurs within a framework of respect for their privacy and autonomy. Some also point out that the therapeutic bond established with professionals facilitates this participation, promoting fluid but non-invasive collaboration.

*“I think it’s fine, privacy is maintained, and when there are questions for my parents, they’re no longer excluded, but it also helps that they’re not always there”* (U2).

Families recognize their active participation and feel welcomed by the care team. However, some primary caregivers claimed to be unaware of the possibility of involving and integrating other meaningful figures into the care process. This lack of information can limit the participation of key actors in care, especially in contexts of high family overload.

From the perspective of professionals, it is recognized that, although the current healthcare model allows for the inclusion of primary caregivers, in practice family participation is often limited to a single figure, due to the widespread lack of family, social, or institutional support faced by most families with a person with autism in their care.

*“Families are very isolated because they cannot leave their child with other caregivers and they have no support from other family members or institutions, and sometimes they themselves lack the resources to understand their children…”* (P3).

It should also be noted that certain legal barriers, such as some of the restrictions imposed by data protection or patient protection laws, make it difficult to establish effective communication with other relevant members of the family or social environment of patients with autism when there is no explicit consent from the data subject. These limitations can restrict collaborative networking and reduce opportunities to offer a broader and more collaborative approach.

*“The problem we encounter when dealing with nursing homes, data protection laws, child protection laws, and patient rights laws…. So, if they want to contact us, but the parent doesn’t want them to, there’s nothing we can do…”* (P4).

#### 3.1.7 Continuity and transition

Continuity of care and effective management of transitions between treatment stages or healthcare settings are critical aspects of care for individuals with ASD. From the patient perspective, continuity with specific professionals with whom a stable therapeutic relationship is established over time is highly valued, particularly when care extends beyond pharmacological treatment to include personalized interventions. However, patients report substantial challenges related to frequent specialist turnover, the absence of replacements during holiday periods, and the lack of a stable clinical reference across the life course, which generate uncertainty and feelings of abandonment at key points in the care pathway.

*“So there hasn’t really been any support in that transition, because it was a different person every time”* (U2).

On the part of families, the perception of continuity of care is very positive, highlighting the clarity of the guidance received after consultations, the availability of the team in the event of therapeutic changes, and the proactive attitude of professionals in managing adjustments to patient interventions. They also particularly emphasize the facilitating role of email contact, which has already been addressed by service users, as well as the two-way nature of clinical decision-making. In this sense, families feel listened to and supported, with sufficient resources to sustain the care of the person with ASD in their home environment, even in contexts of change or adaptation of treatment.

*“In light of all the situations and changes that have arisen, treatment proposals have been made, and follow-up has been carried out, because ultimately we understand that treatments are highly personalized and also very clinical”* (F4).

However, in this area, professionals share a critical view, warning of the existence of a healthcare model that, although it offers adequate and structured support during childhood and adolescence, has significant shortcomings specifically during the transition to adult care. Professionals point to the lack of specific social and health services for adulthood, especially once they leave the educational context, which results in a discontinuity of care, difficulties in managing complex clinical situations, and a heavy burden on families. Furthermore, it is noted that, although specific efforts are made to guide patients and their families (such as explanatory interviews or communications via email), these measures are insufficient to respond to the demands of this stage of change and transition.

*“Also, when they transition from adolescence to adulthood.… It’s not just within the AMITEA program, but when they transition from adolescence to adulthood and from AMITEA to external public mental health services, then it’s chaos because they don’t know what to do” (P1). “I think the transition to adulthood is vital in ASD and non-ASD, and it’s a failure right now” (P6). “On a social level, there’s nothing at all for adults…”* (P9).

#### 3.1.8 Access to care

From the patients’ perspective, the AMITEA unit’s central location and accessibility via public transport are considered adequate, though some features of the surrounding area may pose additional barriers for those with reduced mobility. Regarding infrastructure, improvements such as soundproofing the waiting room are suggested to better accommodate users’ sensory needs. Both patients and families value efficient email communication with the AMITEA team, yet persistent telephone line issues and the lack of direct contact with some professionals generate uncertainty in non-emergency situations requiring timely action.

Professionals note that direct patient contact with the psychiatry department is limited and usually initiated by staff, as the unit is not designed for 24-hour emergency care; emergencies should be directed to hospital services. Scheduled follow-up appointments are emphasized as the most effective channel for addressing clinical needs. Professionals also identify significant barriers due to limited awareness of the AMITEA referral process among primary care, mental health, and other hospital staff, resulting in unnecessary steps that delay access for patients with autism.

*“Not everyone who refers patients with ASD knows AMITEA, so the path that patients take before arriving here is very diverse. Some families find the process very difficult, they don’t know what to do, they follow the instructions of their primary care physicians and those who refer them but often leads them to wasted time…”* (P7).

Experiences vary when it comes to obtaining appointments within AMITEA or referrals to other specialists or external services. While some users highlight the speed with which certain medical needs are addressed, others mention significant delays, especially in specialties such as dentistry or services outside the hospital itself. There is also some confusion about the current role of the service, which can lead to a mismatch between families’ expectations and the team’s actual capacity to intervene, thus compromising the effectiveness of the care process.

*“Speaking a little from the psychology service (…) it should be noted that it is not a typical psychology service such as the one provided by local Mental Health Centre [another public resource]. So, when referrals to the psychology service in AMITEA come in, mainly from our own internal psychiatrists, many parents are fine, but others come with the expectation of a standard psychotherapeutic follow-up, and the initial appointments are sometimes more complicated in terms of expectations from the families”* (P1).

### 3.2 Deep interpretation

Following Ricoeur, the final phase of the hermeneutic arc, which constitutes the culmination of the interpretive process, refers to the process of understanding. This can be defined as an openness to the mode of being-in-the-world to which the text refers.

We now present the results of the interpretation of lived experience derived from the analysis of the lived experience of each of the three groups.

#### 3.2.1 Professionals’ perspective

For the professionals interviewed, the most relevant aspect of the AMITEA program lies in its condition as an instrument, an effective complementary tool that supports the actions of healthcare specialists and facilitates the care of patients with ASD who present special needs. For non-specialized professionals in particular, patients with ASD exhibit specific needs that, despite their willingness, they are unable to address on their own.

It is noteworthy that most of the negative remarks and perceived weaknesses identified by professionals are not directed at the program itself, but rather at characteristics of the healthcare system as a whole and the context in which they work. These include bureaucratic barriers, legal constraints, excessive workload, scarcity of resources, and, in some cases, a lack of awareness or understanding among colleagues.

Professionals express a clear desire to meet the needs of their patients with ASD; however, they are embedded in a reality that prevents them from doing so independently. Within this context, the AMITEA program is perceived as a tool that, to a certain extent, counteracts both the barriers imposed by the healthcare system and those inherent to ASD in the treatment process.

#### 3.2.2 Family members’ perspective

The interviews conducted with family members of AMITEA users reveal an overall positive evaluation of the service. At the same time, this group is the one that identifies the greatest number of negative situations associated with the program, such as insufficient information, lack of sensitivity in external services, or the potential for improvements in service accessibility. It is also illuminating that professionals point out that, at times, families’ expectations are not fully aligned with the program’s actual capacities.

Where does this divergence originate, given that it does not lead to a negative overall assessment of the service (as noted in one interview, the program’s drawbacks are perceived as being offset by its strengths)? The structural analysis highlights that a significant portion of both positive and negative evaluations expressed by family members are accompanied by feelings of concern for their children and by the emotional burden derived from their caregiving role.

For these families, ASD introduces an additional dimension to their condition as caregivers, resulting in increased stress, workload, and disruption of family dynamics. AMITEA represents an opportunity to reduce stress and emotional burden. However, because the program is primarily oriented toward the care of the user, family members may at times perceive that their own needs are not fully addressed, which may constitute an area for future improvement.

#### 3.2.3 Users’ perspective

One concept stands out prominently in the accounts of AMITEA users: the feeling of “being understood.” The interviews reveal the users’ positioning in relation to a world that, beyond their closest references, such as family members or the program itself, appears alien and difficult to engage with communicatively. In this context, feeling understood, and not only that, but also respected, emerges as particularly significant for patients with ASD.

Outside the program, users’ references to various professional services indicate that these operate according to rules and procedures to which patients are expected to adapt, often without being able to understand them, thereby generating a pervasive sense of being lost.

The “external” world is depicted as a Kafkaesque space in which, much like the protagonist of The Castle, users are subjected to a series of processes and restrictions that they cannot fully comprehend and within which their voices are not heard. Within the program, however, this relationship is reversed: not only is the user’s voice taken into account, but the program’s procedures are adapted to the individual patient, rather than requiring the patient to adapt to them.

The lived experience narrated by AMITEA users reflects the tension between the need for support and the risk of excessively paternalistic supervision. In a system where users’ voices are largely disregarded, individuals with ASD face two equally undesirable extremes: abandonment (the inability to access the services needed to meet their needs) or excessive paternalistic control, which guides them blindly through an incomprehensible labyrinth. By contrast, the AMITEA program offers an alternative that addresses users’ needs while respecting their voice, through a form of accompaniment that provides support while preserving autonomy, privacy, and agency.

## 4 Discussion

The results presented here correspond to opinions from a threefold perspective (i.e., users/patients, family members, and professionals), with a qualitative approach, regarding the services offered by a medical unit specializing in the hospital treatment of patients with ASD: the AMITEA unit of the Gregorio Marañón General University Hospital in Madrid (Spain). The results of this study show that the opinions of the different types of interviewees largely coincide on the weaknesses and strengths of this unit, but they also reveal contrasts that allow for a deeper understanding on how to address the healthcare of patients with ASD.

From Ricoeur’s hermeneutic phenomenology, the participants’ analyzed discourse is not limited to describing facts but rather expresses meaningful experiences in which the subject positions themselves in relation to their experience, interprets it, and thus communicates it. This narrative dimension provides access to symbolic structures that shape the way individuals understand their place in the healthcare system, their relationship with professionals, and the impact that the care they receive has on their identity and dignity.

### 4.1 Humanizing and healthcare services

The interpretation of the results collected invites reflection on the current challenge faced by healthcare systems in their transition toward a necessary humanization of their practices (Busch et al., 2019), services (Correa, 2016), and policies (Nora and Junges, 2013), as well as their physical and environmental settings (Bates, 2018). This transformation cannot be understood as a mere technical or aesthetic adjustment, but rather as a comprehensive reconfiguration of the healthcare model, in which the patient is recognized as a subject of rights (Brännmark, 2017). The requirement set forth becomes especially urgent in groups with chronic health conditions, such as ASD (Parker and Killian, 2020), whose care requires not only clinical continuity, but also relational sensitivity, institutional flexibility, and the creation of mechanisms adapted to the patient’s needs throughout their life cycle (Malik-Soni et al., 2022). In these cases, humanization is not consolidated as an added value, but as a condition of possibility for truly effective patient care.

The experience of the participants in this study shows that, when the system manages to articulate person-centered practices, it generates experiences of recognition and belonging to the service, as well as patient safety and wellbeing (Mohammed et al., 2016). The testimonies collected reveal that the care received at AMITEA is perceived as radically different from that offered in other public clinical services, especially when compared to that received in units not specialized in autism care.

Users with ASD participating in the research (with low support needs) highlight, amongst other positive aspects, the quality of the therapeutic bond established between service professionals and the patients themselves. However, the experience is not uniform, and family members of those with greater support needs point out slight deficiencies in the psycho-emotional care as well as in the involvement of support networks. This divergence suggests that the humanization of care, although present, is not distributed equitably, which directly challenges distributive justice in the healthcare setting, especially when referring to those with high support needs (Galiatsatos et al., 2020).

### 4.2 Toward an accessible health system

From a strategic perspective, moving toward the humanization of healthcare services means advancing toward a truly inclusive system, designed to respond to the needs of all individuals without exception (Zandam and Juni, 2019). Regardless of the semantic variations that the concept of “access” may acquire, it constitutes the fundamental starting point for the efficient functioning of any healthcare system. Therefore, addressing access from a multilevel perspective, encompassing both the structural and relational dimensions of the care process, is essential for designing interventions that enable its effective operationalization (Levesque et al., 2013). This approach enables not only formal entry into the system, but also full and equitable enjoyment of health services throughout the patient’s clinical journey.

The participants in this study expressed a highly positive assessment of the sensory adaptation of the physical environment of the specialized AMITEA program, such as the suitability of the waiting rooms and the strategic location of the hospital, which is key due to its accessibility by public transport. Likewise, the data collected shows that accessibility in healthcare services cannot be understood solely from an infrastructural perspective. While these elements are valued positively, relational dimensions that directly affect the healthcare experience are also identified. Participants point out the benefits of the personal accompaniment and speed up attendance in other specialties. The existence of agile communication channels, flexibility in appointment management, and the willingness of staff to accompany and guide users through the service are practices that extend the concept of accessibility beyond the physical or structural. The relational dimension of access is consolidated as an essential component that guarantees equitable care for patients with significant needs (Boada and Parellada, 2017). The benefits derived from these conditions are reflected in a substantial improvement in the user experience, both in terms of satisfaction and clinical outcomes. Reduced uncertainty, fewer critical events in sensitive contexts, and a stronger therapeutic bond are some of the observable effects when accessibility is conceived as a comprehensive practice (Thom et al., 2019). In this sense, services that manage to integrate the structural and relational dimensions of access not only optimize the efficiency of the system, but also promote more humane, person-centered care.

In contrast, the literature reflects also on the consequences of inadequate accessibility. The identification of barriers to accessing healthcare (Carrillo et al., 2011), such as difficulties or delays in the appointment management process, limitations in communication or information processes, and the lack of adaptation of external services (e.g., hospital emergency departments or specialist clinics) is experienced as a situation of exclusion that directly affects the clinical experience of patients and their companions. These types of barriers are critically important in the case of people with ASD, whose sensory sensitivity and need for anticipation require the creation of adapted and predictable clinical environments. However, there is evidence pointing to significant gaps in clinical knowledge about the management of ASD in healthcare settings, which translates into widespread care characterized by fragmentation and a lack of adaptation to the specific needs of these patients (Morris et al., 2019), an experience pointed out in the focus groups.

In this regard, our findings coincide with the review by Dern and Sappok (2016), who identified a list of key factors considered to be obstacles in the healthcare processes for patients with ASD, including, among others, the aforementioned difficulties in making appointments, the sensory overload characteristic of waiting rooms in healthcare centers, communication barriers between patients and healthcare professionals, and the rigidity of clinical procedures that these types of users and their families frequently face. The repeated experience of these barriers by users and family members challenges the healthcare system’s ability to recognize neurodiversity as a legitimate part of the human condition and, therefore, of healthcare (Scott et al., 2021).

### 4.3 Improving coordination of care

Interprofessional, interlevel, and interservice referral practices are associated with higher-quality healthcare processes (Elliott et al., 2021). The coordination protocols established within the AMITEA model are considered one of the pillars of the service, positioning psychiatrists, but also specialized nursing professionals, as key agents in the coordination of care for patients with ASD and guarantors of continuity of care and build strategic bridges between the different clinical specialties for patients with ASD. In this sense, in line with the findings of Parker and Killian (2020), AMITEA specialist nurses play an ideal role that allows them, based on a comprehensive view of the patient and their family, to respond effectively to the complex and chronic needs arising from autism.

Although users and families underscore that the principal strength lies in the coordination initiated by specialist nurses and clinicians with both healthcare and non-healthcare facilities, the persistence of structural barriers in non-specialized clinical services compromises the effectiveness of an integrative care model, notably the absence of standardized clinical protocols between services, the lack of systems for early identification of patients with ASD in care circuits, and the difficulty in establishing two-way communication channels between different levels of healthcare. The heterogeneity in procedures and criteria for action among different clinical services not only hinders the continuity of care for people with autism but also has a direct impact on the quality of care provided to patients. To move toward effective interprofessional collaboration, it is essential to consolidate shared standards that facilitate joint work between the health disciplines most involved in the clinical intervention of autism (Bowman et al., 2021). Likewise, this coordination must extend beyond the clinical setting, incorporating mechanisms for cooperation with other key sectors in the patient’s life, such as education and social services, whose integration is essential to ensure truly person-centered care (Gardner et al., 2022). Thus, mechanisms to enhance coordination and care in services and settings beyond AMITEA should be prioritized.

### 4.4 Understanding Autism through family experience

The participation of family members and primary caregivers in the care process for people with ASD is a fundamental part of providing healthcare that is both continuous over time and effective (Factor et al., 2019). Families not only act as mediators between the patient and the system, but also take on roles of clinical support, emotional management, and care coordination, especially in highly complex contexts (Parker and Killian, 2020). However, as revealed in this study, this involvement is significantly conditioned by structural limitations that restrict their active participation, such as the lack of institutional support or difficulties in coordination between healthcare professionals and other sectors in cases where families do not allow direct access to their health data. The results of the research show that this situation creates a feeling of isolation among primary caregivers, who face a significant emotional burden in the absence of formalized support networks.

Specifically, the discourses collected in the research reflect that, although childhood and adolescence are usually accompanied by more accessible and specialized clinical services and social resources, the transition to adulthood represents a break in continuity of care. This discontinuity translates into a lack of stable clinical references, leading to the progressive invisibility of patients with ASD in healthcare systems, but also in social settings. As a result, families experience profound feelings of abandonment and overload, especially caregivers who are also going through their own aging process (Oti-Boadi et al., 2020). This experience of loss of meaning, both clinically and socially, generates substantial structural inequality compared to previous stages in the life cycle of people with autism (Ee et al., 2019).

The complexity inherent in adulthood, coupled with a lack of coordination between health services, creates a scenario of vulnerability that requires deliberate institutional responses. As Pickler and Dressler (2022) point out, the absence of structured transition mechanisms and specific policies for the care of adults with ASD creates barriers that limit effective access to health services. Overcoming these limitations requires not only a reconfiguration of care models, but also a critical review of inclusion policies to ensure that people with ASD and their families can navigate the health system without experiencing discontinuities that compromise their wellbeing and their right to equitable care. In this regard, it is particularly relevant to highlight the experience of AMITEA, whose “0–99 years” care model constitutes one of its fundamental pillars to ensure continuity of care in medical processes requiring hospitalization. This approach demonstrates that the existence of adapted models can help reduce gaps in healthcare, while also underscoring the need to strengthen communication channels with families and users themselves, so that they can fully understand the scope and mission of these services, thus fostering more effective access and a safer experience within the healthcare system. Finally, Table 2 shows Recommendations for the humanization of healthcare services based on our findings.

**TABLE 2:** Recommendations for the humanization of healthcare services based on our findings.

Recommendation	Description and rationale based on study findings
1. Institutionalize recognition as the starting point of care *(Picker’s Principle: Respect for patients’ values, preferences, and expressed needs)*	Health services should be designed so that care begins with naïve understanding of the patient with ASD as a meaning-bearing subject. Clinical encounters must prioritize listening, adapted communication, and respect for individual preferences before diagnostic or procedural explanation. This enables a form of care in which recognition and dignity are conditions of possibility for effective intervention and not just outcomes.
2. Design ASD services as mediating structures rather than isolated units *(Picker’s Principle: Coordination and integration of care)*	Healthcare systems should function as hermeneutic mediators, actively translating between different professional, institutional, and social “worlds.” Dedicated coordinating roles and shared protocols should be established to ensure that explanation across services supports, rather than fragments, the person with ASD’s lived experience of care.
3. Ensure intelligibility of care processes through autistic-friendly adapted explanation *(Picker’s Principle: Information and education)*	Information provided to people with ASD and their carers should not merely transmit facts, but support comprehension. Care pathways must be predictable, anticipatory, and tailored to neurodiverse ways of understanding, enabling patients and families to appropriate information meaningfully and navigate the system with confidence and ease.
4. Integrate sensory and environmental adaptation as a main ethical practice *(Picker’s Principle: Physical comfort)*	Clinical environments should be intentionally designed to support an ASD-embodied experience, recognizing sensory adaptation as part of ethical explanation rather than ancillary accommodation. Predictable, low-stimulus environments will enable patients to experience healthcare spaces as safe and intelligible worlds.
5. Embed emotional safety as a core dimension of clinical care *(Picker’s Principle: Emotional support and alleviation of fear and anxiety)*	Healthcare practices should explicitly address the very diverse autistic affective experience, recognizing that emotional safety emerges when understanding and explanation are held in balance. Training and organizational cultures must support relational care that reduces anxiety and fosters trust within specialist ASD understanding, particularly in highly stressful clinical contexts
6. Formally recognize families as interpretive partners in ASD care *(Picker’s Principle: Involvement of family and friends)*	Families should be integrated as co-interpreters of the care process, whose narratives and knowledge contribute to meaningful understanding of the patient’s very unique needs. Services must move beyond informal reliance on caregivers toward structured inclusion and institutional recognition of their role.
7. Safeguard narrative continuity across the life course *(Picker’s Principle: Continuity and transition)*	Healthcare systems must preserve narrative coherence by ensuring stable clinical references and structured transitions, particularly from adolescence to adulthood. Lifespan-oriented models of care allow individuals with ASD to maintain continuity of identity and meaning within the healthcare system.
8. Conceive access as a basic, lived and relational experience *(Picker’s Principle: Access to care)*	Access to care should be understood not only as physical or administrative entry but as a relational experience shaped by responsiveness, accompaniment, and adequate communication. Flexible scheduling and accessible contact channels enable ASD patients to experience themselves as held within, rather than excluded from, the healthcare system.

### 4.5 Limitations and future directions

This study has methodological limitations that should be considered when interpreting the results. The patient sample was restricted exclusively to individuals with functional verbal communication skills, which excludes a significant portion of the ASD clinical spectrum. To mitigate this limitation, family members of patients with additional intellectual disabilities were deliberately included to ensure a broader representation of users’ experiences. However, while caregivers’ perspectives provide valuable insights, they cannot replace the lived experience or first-person perspectives of autistic individuals themselves. This limitation reflects a broader methodological challenge in autism research, where established qualitative approaches often struggle to adequately capture the perspectives of autistic individuals with higher support needs. Therefore, it is imperative to further amplify the voice of these individuals to advocate for themselves, and future studies should prioritize the development and adaptation of methodologies that enable their meaningful inclusion and cover their unique needs.

Moreover, the autistic participants were young adults and most of the caregivers were parents of adults, so the perspectives presented here may largely relate to adult care pathways. However, most adult participants had been involved in the program since childhood or adolescence, which allows a reflection on experiences across different life-stages, and two of the eight caregivers were parents of children, likewise enabling a broad perspective within the focus group discussion. On another note, the duration of the group sessions (90 min) may have limited the exploration of relevant dimensions such as economic implications, types of medical needs, or services used, among other relevant factors. To complement this information, the research team is currently developing a systematic data collection process using anonymous online questionnaires aimed at program users, which will allow other care priorities to be addressed and the sample to be significantly expanded.

The findings from this study underscore the value of patient-centered practices in providing care to underserved populations, specifically people with ASD and their caregivers. Lived experiences of patients, caregivers and professionals highlight the advantages of taking steps to humanizing the healthcare process in hospital settings and reveal the relevance of implementing not only environmental adaptations but also transforming structural and systemic practices to pursue and promote accessibility, equity and empathy in the healthcare context.

## Data Availability

The raw data supporting the conclusions of this article will be made available by the authors, without undue reservation.
