# Supplementary material for: Humanizing medical care for individuals with autism spectrum disorder and their families: the experience of healthcare support in the Comprehensive Medical Care Unit for individuals with ASD (AMITEA)
Source: Front Psychol. 2026 Feb 11;17:1716298. doi: 10.3389/fpsyg.2026.1716298 (PMC12932931; doi:10.3389/fpsyg.2026.1716298)
Supplement: Supplementary file 2 [file Data_Sheet_2.pdf]

FOCUS GROUP FAMILIES

1. Respect for expressed values, preferences, and needs:

To what extent do you feel that the medical team takes into account the values, beliefs, or preferences of the family/patient in the care of the person with ASD?

Have you felt listened to and understood during the care process?

Have there been situations in which the autonomy or decisions of the patient's family members have not been respected?

2. Coordination and integration of care:

How would you describe the coordination between the different professionals involved in the care (doctors, therapists, social workers)?

Have you had difficulties coordinating medical and support services?

Do you consider that there is fluid communication between the different levels or specialties of the healthcare system?

3. Information and education:

Did you receive clear and sufficient information about the diagnosis, progression, and treatment?

Does the information provided allow you to make informed decisions?

What type of guidance or materials have you found most useful? What information have you found lacking?

4. Physical comfort:

How do you rate the attention given to physical needs during medical visits or stays?

Do you consider the physical environment (spaces, waiting rooms, sensory stimuli, etc.) to be adapted to the characteristics of people with ASD?

Are situations of discomfort, pain, or crisis during consultations or treatments managed appropriately?

5. Emotional support and relief from fear and anxiety:

Have you felt emotional support from healthcare staff?

Is the team sensitive to family stress, concerns, or exhaustion?

Are there spaces or professionals to help manage anxiety or emotions during the care process?

6. Involvement of family and friends:

Have they been actively involved in care-related decisions?

Do they feel that their role as caregivers is valued and supported?

How is the participation of other family members in care facilitated or hindered?

7. Continuity and transition:

Did you receive clear guidance on the steps to take after medical consultations or hospital stays?

Did you feel prepared to continue care at home?

How would you rate the support provided during transitions between stages of treatment or between different services?

8. Accessibility to healthcare:

How easy has it been to access the unit (location, transportation, hours, contact)?

Have you had difficulty getting appointments or referrals when you needed them?

Do you consider that the system is adapted to the specific needs of people with ASD and their families?

Closing

Is there any other aspect related to specialized healthcare that you would like to share?

What improvements do you consider a priority to enhance the care experience?

FOCUS GROUP PARTICIPANTS

1. Respect for expressed values, preferences, and needs:

To what extent do you feel that the medical team takes your values, beliefs, or preferences into account in your care?

Have you felt listened to and understood during the care process?

Have there been situations in which your autonomy or decisions have not been respected?

2. Coordination and integration of care:

How would you describe the coordination between the different professionals involved in your care (doctors, therapists, social workers)?

Have you had difficulty coordinating your medical and support services?

Do you feel there is fluid communication between the different levels or specialties of the healthcare system?

3. Information and education:

Did you receive clear and sufficient information about your diagnosis, progress, and treatment?

Does the information provided to you allow you to make informed decisions?

What type of guidance or materials have you found most useful? What information have you found lacking?

4. Physical comfort:

How would you rate the attention given to physical needs during medical visits or stays?

Do you consider the physical environment (spaces, waiting rooms, sensory stimuli, etc.) to be adapted to the characteristics of people with ASD?

Are situations of discomfort, pain, or crisis during consultations or treatments managed appropriately?

5. Emotional support and relief from fear and anxiety:

Have you felt emotionally supported by healthcare staff?

Is the team sensitive to your stress, concerns, or exhaustion?

Are there spaces or professionals who help you manage your anxiety or emotions during the care process?

6. Involvement of family and friends:

Have they been actively involved in decisions related to your care?

Do you feel that their role as caregivers is valued and supported?

How is the involvement of other family members in your care facilitated or hindered?

7. Continuity and transition:

Did you receive clear guidance on the steps to take after medical appointments or hospital stays?

Did you feel prepared to continue care at home?

How do you rate the support provided during transitions between stages of treatment or between different services?

8. Accessibility to healthcare:

How easy has it been to access the unit (location, transportation, hours, contact)?

Have you had difficulty getting appointments or referrals when needed?

Do you think the system is adapted to the specific needs of people with ASD and their families?

Closing

Is there anything else related to specialized healthcare that you would like to share?

What improvements do you consider a priority to enhance the care experience?

PROFESSIONAL FOCUS GROUP

1. Respect for expressed values, preferences, and needs:

To what extent do you feel that the medical team takes into account the values, beliefs, or preferences of the family/patient in the care of the person with ASD?

Have you felt listened to and understood during the care process?

Have there been situations in which the autonomy or decisions of the family/patient have not been respected?

2. Coordination and integration of care:

How would you describe the coordination between the different professionals involved in the care (doctors, therapists, social workers)?

Have you had difficulties coordinating medical and support services?

Do you consider that there is fluid communication between the different levels or specialties of the healthcare system?

3. Information and education:

Did you receive clear and sufficient information about the diagnosis, progression, and treatment?

Does the information provided allow you to make informed decisions?

What type of guidance or materials have you found most useful? What information have you found lacking?

4. Physical comfort:

How would you rate the attention given to physical needs during medical visits or stays?

Do you consider the physical environment (spaces, waiting rooms, sensory stimuli, etc.) to be adapted to the characteristics of people with ASD?

Are situations of discomfort, pain, or crisis during consultations or treatments handled appropriately?

5. Emotional support and relief from fear and anxiety:

Have you felt emotional support from healthcare staff?

Is the team sensitive to family stress, concerns, or exhaustion?

Are there spaces or professionals who help manage anxiety or emotions during the care process?

6. Involvement of family and friends:

Have they been actively involved in care-related decisions?

Do they feel that their role as caregivers is valued and supported?

How is the participation of other family members in care facilitated or hindered?

7. Continuity and transition:

Did they receive clear guidance on the steps to take after medical appointments or hospital stays?

Did they feel prepared to continue care at home?

How do they rate the support provided during transitions between stages of treatment or between different services?

8. Accessibility of healthcare:

How easy has it been to access the unit (location, transportation, hours, contact)?

Have you had difficulty getting appointments or referrals when you needed them?

Do you think the system is adapted to the specific needs of people with ASD and their families?

Closing

Is there anything else related to healthcare that you would like to share?

What improvements do you consider a priority to enhance the care experience?
