# Supplementary material for: Humanizing medical care for individuals with autism spectrum disorder and their families: the experience of healthcare support in the Comprehensive Medical Care Unit for individuals with ASD (AMITEA)
Source: Front Psychol. 2026 Feb 11;17:1716298. doi: 10.3389/fpsyg.2026.1716298 (PMC12932931; doi:10.3389/fpsyg.2026.1716298)
Supplement: Supplementary file 3 [file Data_Sheet_3.pdf]

FOCUS GROUP FAMILIAS (Original version)

1. Respeto por los valores, preferencias y necesidades expresadas:

¿En qué medida sienten que el equipo médico tiene en cuenta los valores, creencias o preferencias de la familia/el paciente en el cuidado de la persona con TEA?

¿Se han sentido escuchados y comprendidos durante el proceso de atención?

¿Ha habido situaciones en las que no se haya respetado la autonomía o las decisiones de los familiares del paciente?

2. Coordinación e integración de la atención:

¿Cómo describen la coordinación entre los distintos profesionales que intervienen en la atención (médicos, terapeutas, trabajadores sociales)?

¿Han tenido dificultades para coordinar los servicios médicos y de apoyo?

¿Consideran que existe una comunicación fluida entre los diferentes niveles o especialidades del sistema sanitario?

3. Información y educación:

¿Recibieron información clara y suficiente sobre el diagnóstico, evolución y tratamiento?

¿La información que se les proporciona permite tomar decisiones informadas?

¿Qué tipo de orientación o materiales les han resultado más útiles? ¿Qué información han echado en falta?

4. Comodidad física:

¿Cómo valoran la atención a las necesidades físicas durante las visitas o estancias médicas?

¿Consideran que el entorno físico (espacios, salas de espera, estímulos sensoriales, etc.) está adaptado a las características de las personas con TEA?

¿Se gestionan adecuadamente situaciones de incomodidad, dolor o crisis durante las consultas o tratamientos?

5. Apoyo emocional y alivio del miedo y la ansiedad:

¿Han sentido apoyo emocional por parte del personal sanitario?

¿El equipo muestra sensibilidad ante el estrés, las preocupaciones o el agotamiento familiar?

¿Existen espacios o profesionales que ayuden a gestionar la ansiedad o las emociones durante el proceso asistencial?

6. Participación de familiares y amigos:

¿Han sido implicados activamente en las decisiones relacionadas con el cuidado?

¿Sienten que su papel como cuidadores es valorado y apoyado?

¿Cómo se facilita o se dificulta la participación de otros miembros de la familia en la atención?

7. Continuidad y transición:

¿Recibieron orientación clara sobre los pasos a seguir después de las consultas o estancias médicas?

¿Se sintieron preparados para continuar con los cuidados en casa?

¿Cómo valoran el acompañamiento en las transiciones entre etapas del tratamiento o entre diferentes servicios?

8. Accesibilidad a la atención sanitaria:

¿Cómo de fácil ha sido acceder a la unidad (ubicación, transporte, horarios, contacto)?

¿Han tenido dificultades para conseguir citas o derivaciones cuando lo han necesitado?

¿Consideran que el sistema se adapta a las necesidades específicas de las personas con TEA y sus familias?

Cierre

¿Hay algún otro aspecto relacionado con la atención sanitaria especializada que quieran compartir?

¿Qué mejoras consideran prioritarias para mejorar la experiencia de atención?

FOCUS GROUP PARTICIPANTES

1. Respeto por los valores, preferencias y necesidades expresadas:

¿En qué medida sientes que el equipo médico tiene en cuenta tus valores, creencias o preferencias en tu cuidado?

¿Te has sentido escuchado/a y comprendido/a durante el proceso de atención?

¿Ha habido situaciones en las que no se haya respetado tu autonomía o tus decisiones?

2. Coordinación e integración de la atención:

¿Cómo describes la coordinación entre los distintos profesionales que intervienen en tu atención (médicos, terapeutas, trabajadores sociales)?

¿Has tenido dificultades para coordinar tus servicios médicos y de apoyo?

¿Consideras que existe una comunicación fluida entre los diferentes niveles o especialidades del sistema sanitario?

3. Información y educación:

¿Recibiste información clara y suficiente sobre tu diagnóstico, evolución y tratamiento?

¿La información que se te proporciona te permite tomar decisiones informadas?

¿Qué tipo de orientación o materiales te han resultado más útiles? ¿Qué información has echado en falta?

4. Comodidad física:

¿Cómo valoras la atención a las necesidades físicas durante las visitas o estancias médicas?

¿Consideras que el entorno físico (espacios, salas de espera, estímulos sensoriales, etc.) está adaptado a las características de las personas con TEA?

¿Se gestionan adecuadamente situaciones de incomodidad, dolor o crisis durante las consultas o tratamientos?

5. Apoyo emocional y alivio del miedo y la ansiedad:

¿Has sentido apoyo emocional por parte del personal sanitario?

¿El equipo muestra sensibilidad ante tu estrés, preocupaciones o agotamiento?

¿Existen espacios o profesionales que te ayuden a gestionar tu ansiedad o tus emociones durante el proceso asistencial?

6. Participación de familiares y amigos:

¿Han sido implicados activamente en las decisiones relacionadas con tu cuidado?

¿Sientes que su papel como cuidadores es valorado y apoyado?

¿Cómo se facilita o se dificulta la participación de otros miembros de la familia en tu atención?

7. Continuidad y transición:

¿Recibieron orientación clara sobre los pasos a seguir después de las consultas o estancias médicas?

¿Se sintieron preparados para continuar con los cuidados en casa?

¿Cómo valoran el acompañamiento en las transiciones entre etapas del tratamiento o entre diferentes servicios?

8. Accesibilidad a la atención sanitaria:

¿Cómo de fácil ha sido acceder a la unidad (ubicación, transporte, horarios, contacto)?

¿Has tenido dificultades para conseguir citas o derivaciones cuando lo han necesitado?

¿Consideras que el sistema se adapta a las necesidades específicas de las personas con TEA y sus familias?

Cierre

¿Hay algún otro aspecto relacionado con la atención sanitaria especializada que quieras compartir?

¿Qué mejoras consideras prioritarias para mejorar la experiencia de atención?

FOCUS GROUP PROFESIONALES

1. Respeto por los valores, preferencias y necesidades expresadas:

¿En qué medida sienten que el equipo médico tiene en cuenta los valores, creencias o preferencias de la familia/el paciente en el cuidado de la persona con TEA?

¿Se han sentido escuchados y comprendidos durante el proceso de atención?

¿Ha habido situaciones en las que no se haya respetado la autonomía o las decisiones familiares/del paciente?

2. Coordinación e integración de la atención:

¿Cómo describen la coordinación entre los distintos profesionales que intervienen en la atención (médicos, terapeutas, trabajadores sociales)?

¿Han tenido dificultades para coordinar los servicios médicos y de apoyo?

¿Consideran que existe una comunicación fluida entre los diferentes niveles o especialidades del sistema sanitario?

3. Información y educación:

¿Recibieron información clara y suficiente sobre el diagnóstico, evolución y tratamiento?

¿La información que se les proporciona permite tomar decisiones informadas?

¿Qué tipo de orientación o materiales les han resultado más útiles? ¿Qué información han echado en falta?

4. Comodidad física:

¿Cómo valoran la atención a las necesidades físicas durante las visitas o estancias médicas?

¿Consideran que el entorno físico (espacios, salas de espera, estímulos sensoriales, etc.) está adaptado a las características de las personas con TEA?

¿Se gestionan adecuadamente situaciones de incomodidad, dolor o crisis durante las consultas o tratamientos?

5. Apoyo emocional y alivio del miedo y la ansiedad:

¿Han sentido apoyo emocional por parte del personal sanitario?

¿El equipo muestra sensibilidad ante el estrés, las preocupaciones o el agotamiento familiar?

¿Existen espacios o profesionales que ayuden a gestionar la ansiedad o las emociones durante el proceso asistencial?

6. Participación de familiares y amigos:

¿Han sido implicados activamente en las decisiones relacionadas con el cuidado?

¿Sienten que su papel como cuidadores es valorado y apoyado?

¿Cómo se facilita o se dificulta la participación de otros miembros de la familia en la atención?

7. Continuidad y transición:

¿Recibieron orientación clara sobre los pasos a seguir después de las consultas o estancias médicas?

¿Se sintieron preparados para continuar con los cuidados en casa?

¿Cómo valoran el acompañamiento en las transiciones entre etapas del tratamiento o entre diferentes servicios?

8. Accesibilidad a la atención sanitaria:

¿Cómo de fácil ha sido acceder a la unidad (ubicación, transporte, horarios, contacto)?

¿Han tenido dificultades para conseguir citas o derivaciones cuando lo han necesitado?

¿Consideran que el sistema se adapta a las necesidades específicas de las personas con TEA y sus familias?

Cierre

¿Hay algún otro aspecto relacionado con la atención sanitaria que quieran compartir?

¿Qué mejoras consideran prioritarias para mejorar la experiencia de atención?
